# Synergistic activity of ceftazidime/avibactam combined with aztreonam against MBL-producing *exoY+/exoT+/exoU+/exoS-* extensively drug-resistant *Pseudomonas aeruginosa*

**DOI:** 10.3389/fcimb.2026.1737414

**Published:** 2026-02-27

**Authors:** Xianzhen Wei, Mingbo Liu, Runxian Tan, Peng Huang, Xia Fang, Shan Li, Meng Li

**Affiliations:** 1 Department of Clinical Laboratory, The First People’s Hospital of Qinzhou, Qinzhou, Guangxi, China; 2 Department of Clinical Laboratory, The First Affiliated Hospital of Guangxi Medical University, Nanning, Guangxi, China; 3 Key Laboratory of Clinical Laboratory Medicine of Guangxi Department of Education, Nanning, Guangxi, China

**Keywords:** combined antimicrobial susceptibility testing, extensively drug-resistant *Pseudomonas aeruginosa*, MLST, resistance mechanisms, virulence genes

## Abstract

**Purpose:**

Extensively drug-resistant *Pseudomonas aeruginosa* (XDR-PA) has posed a great threat to public health due to their rising incidence and complicated resistance mechanisms and limited treatment options. XDR-PA has demonstrated high resistance rate to new antibiotic ceftazidime-avibactam (CZA). Therefore, this study was conducted to describe the resistance mechanisms, molecular epidemiology, and type III secretion system (T3SS) of XDR-PA, as well as to evaluate the synergistic antibacterial activity of CZA combined with aztreonam (ATM) against XDR-PA via *in vitro* experiments, aiming at providing insights for the prevention, control and treatment strategies of XDR-PA infections.

**Methods:**

The carbapenemase resistance genes (VIM, IMP, NDM, KPC, GES, OXA-40) and T3SS virulence genes of XDR-PA isolates were identified using polymerase chain reaction (PCR) and sequencing. The expression levels of efflux pump systems (*mexA and mexC*), *oprD2* porin and *ampC* were detected by the real-time fluorescent quantitative PCR (qPCR). The homology analysis of XDR-PA isolates was performed using multilocus sequence typing (MLST). Combined antimicrobial susceptibility testing of CZA and ATM were performed for XDR-PA isolates through *in vitro* experiments.

**Results:**

A total of 32 XDR-PA strains were isolated from clinical specimens from a tertiary teaching hospital in Southwest China between October 2022 to October 2023. Among the carbapenemase detected, metallo-β-lactamase (MBL) NDM-1 and VIM-2 were detected in 26 strains (81.25%, 26/32) and 1 strain (3.13%, 1/32) respectively. The efflux pump *mexA* had a higher expression in the XDR-PA group than that in the sensitive-PA (S-PA) group (*P* = 0.015). The T3SS virulence genes carried by XDR-PA strains mainly were *exoY+/exoT+/exoU+/exoS-* (87.50%, 28/32). The 32 XDR-PA isolates belonged to 8 different ST types, mainly including ST1971 and ST308, and the predominant ST type was ST1971 (71.88%, 23/32), with carrying both NDM-1 and *exoY+/exoT+/exoU+/exoS-*. Combined antimicrobial susceptibility testing revealed that among the 27 CZA-resistant XDR-PA strains, CZA and ATM combination showed a synergistic effect on 21 CZA-resistant XDR-PA strains (77.78%, 21/27), of which 20 strains carrying both MBL (95.24%, 20/21) and *exoY+/exoT+/exoU+/exoS-*.

**Conclusion:**

The underlying resistance mechanisms of XDR-PA isolates involve the overexpression of efflux pump *mexA* and the existence of MBL. In addition, ST1971 was the predominant ST type in our study, with carrying both NDM-1 and *exoY+/exoT+/exoU+/exoS-*. Furthermore, combined antimicrobial susceptibility testing suggested that CZA and ATM combination has potential against MBL-producing *exoY+/exoT+/exoU+/exoS-* XDR-PA. These findings may provide clues for the prevention, control and treatment strategies of XDR-PA infections.

## Introduction

Extensively drug-resistant *Pseudomonas aeruginosa* (XDR-PA) is a great threat to public health due to its rising incidence, limited treatment options, high mortality rate and scarce data on optimal therapy (Horcajada et al., 2019; Del Barrio-Tofiño et al., 2020). Indeed, the incidence rate of XDR-PA ranging from 2.6 to 30.0% had been reported (Walkty et al., 2017; Sader et al., 2018; Horcajada et al., 2019; Pérez et al., 2019). Even though PA is a common cause of respiratory tract infection, urinary tract infection, bloodstream infection and other nosocomial infections, XDR-PA infections are associated with higher mortality (Horcajada et al., 2019). A meta-analysis reported a mortality rate of 62.5% among bacteremia patients infected with XDR-PA versus 30% in those with susceptible PA infections (Recio et al., 2018). Indeed, PA is highly adaptable and can survive in various ecological settings. Its genetic plasticity grants access to many metabolic pathways and a broad range of virulence and resistance factors, making it one of the most successful bacteria in clinical microorganism (Behzadi et al., 2022). The underlying mechanism of XDR-phenotype is heterogeneous and can be mediated through the chromosomal mutations or the horizontal transfer of resistance factors, particularly those encoding carbapenemases often co-transferred with aminoglycoside-modifying enzyme (Del Barrio-Tofiño et al., 2019). These resistance components are closely related to the global high-risk clones, such as ST111, ST235 and ST175 (Oliver et al., 2015). It is known that acquiring antimicrobial resistance often comes with a fitness cost that reduces virulence and disease severity (Gómez-Zorrilla et al., 2016). However, some XDR-PA strains have been linked to the specific virulence factors such as the T3SS *exoU (*Peña et al., 2015; Horna and Ruiz, 2021a). Indeed, PA possesses a virulence mechanism known as the T3SS. The T3SS injects cytotoxins, including *exoU, exoS, exoT* and *exoY*, into target eukaryotic cells, and each of these distinct virulence factors leads to a specific type of host tissue injury, with *exoU* being characterized as having a greater impact on bacterial virulence (Peña et al., 2015; Horna and Ruiz, 2021a).

The XDR-phenotype significantly limits treatment options, although the combination of CZA and ATM has demonstrated promising activity against *Enterobacterales* producing serine-β-lactamase and MBL, it remains uncertain whether this combination is still effective against XDR-PA due to the complex mechanisms underlying the XDR-phenotype (Biagi et al., 2019). Therefore, our study characterized the resistance mechanisms, virulence factors, and molecular epidemiology of XDR-PA, and further evaluated the synergistic antibacterial activity of CZA and ATM combination against XDR-PA, aimed at providing clues for the prevention, control and treatment strategies of XDR-PA infections.

## Materials and methods

### Bacterial isolation and identification

The XDR-PA strains were isolated from a tertiary teaching hospital in Southwest China between October 2022 to October 2023. The sources of the specimens mainly included urine, sputum, secretions, blood and drainage fluid. The study was approved by the Ethics Committee of the First Affiliated Hospital of Guangxi Medical University (Approval Number: 2024-E235-01). The XDR-PA isolates were identified using the VITEK2 Compact system (bioMérieux, Marcy l’Etoile, France) or matrix-assisted laser desorption/ionization time-of-flight mass spectrometry (MALDI-TOF MS) (Autof ms1000, Zhengzhou Antu Biological Engineering Co., LTD). XDR-PA is defined as PA non-susceptibility to at least one agent in all but two or fewer antimicrobial categories, multidrug-resistant (MDR) is defined as non-susceptibility to at least one agent in three or more antimicrobial categories (Magiorakos et al., 2012).

### Antimicrobial susceptibility tests

Antimicrobial susceptibility tests were conducted by the VITEK2 Compact system or the Kirby–Bauer disk diffusion method, except for polymyxin, which was performed with broth microdilution testing. The results were interpreted based on the guidelines of the Clinical and Laboratory Standards Institute (CLSI), version 2025 (Clinical and Laboratory Standards Institute, 2025), besides cefoperazone/sulbactam was interpreted according to the relevant literature (Jones et al., 1987). *Pseudomonas aeruginosa* (ATCC27853) and *Escherichia coli* (ATCC25922) were used as the quality control strains.

### Detection of carbapenemase resistance genes and virulence genes

The carbapenemase resistance genes (NDM, IMP, VIM, KPC, GES, OXA-40) and virulence genes (*exoY, exoT, exoU, exoS*) were identified by PCR and sequenced. The sequencing results were compared with those available in the National Center for Biotechnology Information (NCBI) GenBank database (https://blast.ncbi.nlm.nih.gov/).

### Efflux pump systems, *oprD2* and *ampC* detection

The efflux pump systems (*mexA* and *mexC*), *oprD2*, *ampC* and internal reference genes (RPSL) were detected by qPCR, and *P. aeruginosa* PAO1 was used as the reference strain.

### Multilocus sequence typing

ST types of XDR-PA strains were conducted using seven conserved housekeeping genes (*acsA, guaA, aroE, mutL, ppsA, nuoD* and *trpE*) according to the protocol on the MLST website (https://pubmlst.org/organisms/*pseudomonas-aeruginosa*/primers). The positive amplification products of housekeeping genes were sequencing and compared with PubMLST database (https://pubmlst.org/) to obtain distinct alleles and specific ST type.

### Combined antimicrobial susceptibility testing

Combined antimicrobial susceptibility testing of CZA and ATM were performed for XDR-PA strains using checkerboard broth microdilution. CZA and ATM were separately subjected to 2-fold serial dilution and dispensed into a 96-well microtiter plate, respectively. The bacterial suspension was added into the cation-adjusted Mueller-Hinton broth (CAMHB) to a final concentration of 5 × 10^5^ CFU/mL. Following incubation at 37 °C for 16–18 hours, the minimum inhibitory concentration (MIC) values were determined. The experiments were performed in triplicate. The combination effects were evaluated using the fractional inhibitory concentration index (FIC): FIC = (MIC of drug ATM in the combination/MIC of drug ATM alone) + (MIC of drug CZA in the combination/MIC of drug CZA alone). The results can be divided into four categories:FIC ≤ 0.5, synergistic effect; 0.5 < FIC ≤ 1, additive effect; 1 < FIC ≤ 2, irrelevant effect; FIC > 2, antagonistic effect (Elion et al., 1954).

### Statistical analysis

Statistical analyses were performed using GraphPad Prism version 8.4.3 and SPSS version 23.0. Categorical variables were analyzed using the Chi-squared test or two-tailed Fisher^’^s exact test. Normal distribution continuous variables were expressed as mean ± standard deviation and were analyzed using *t*-test. Continuous variables that were non-normal distributions were expressed as median (interquartile range [IQR]) (using the Mann-Whitney *U*-test). *P* < 0.05 was considered statistically significant.

## Results

### Bacterial isolation and antimicrobial susceptibility tests

A total of 32 XDR-PA strains were isolated, and 32 S-PA strains were included as the control group. The median age of patients infected with XDR-PA was 58.50 (48.25 - 68.75) years old, with 21 males (65.63%, 21/32) and 11 females (34.37%, 11/32). The specimen sources mainly included urine (68.75%, 22/32), sputum (9.38%, 3/32), secretions (9.38%, 3/32) and drainage fluid (9.38%, 3/32). As shown in **Table 1**. The 32 XDR-PA isolates showed resistance to most tested antibiotics except polymyxin. Besides, 84.38% (27/32) XDR-PA isolates showed resistance to CZA (**Table 2**). The antimicrobial susceptibility tests results are listed in **Supplementary Table S1**.

**Table 1:** Demographic and clinical characteristics of patients infected with XDR-PA.

ID	Specimen source	Age (years)/sex	Underlying diseases	LOS (days)	Outcome
PA1	Drainage fluid	52/M	Diabetes, hypertension, pulmonary disease, hepatobiliary disease, kidney disease	53	Automatic discharge
PA2	Secretion	27/M	–	84	Cured
PA3	Sputum	69/M	Cardiac disease, pulmonary disease	67	Cured
PA4	Urine	54/M	–	10	Cured
PA5	Secretion	57/M	Hypertension	15	Cured
PA6	Urine	60/F	Hepatobiliary disease	20	Cured
PA7	Urine	82/M	Cardiac disease, hepatobiliary disease, kidney disease, malignant tumor	16	Cured
PA8	Urine	67/M	Pulmonary disease, kidney disease	24	Cured
PA9	Urine	47/M	Kidney disease, malignant tumor	21	Cured
PA10	Urine	67/M	Hypertension, malignant tumor	10	Cured
PA11	Drainage fluid	62/M	Diabetes, hypertension, cardiac disease, pulmonary disease, hepatobiliary disease, kidney disease	153	Cured
PA12	Urine	46/F	Hepatobiliary disease, kidney disease	21	Cured
PA13	Urine	55/M	Diabetes, kidney disease	15	Cured
PA14	Blood	64/M	Pulmonary disease, hepatobiliary disease, kidney disease	18	Cured
PA15	Drainage fluid	53/F	Hypertension, kidney disease	17	Cured
PA16	Secretion	74/M	Cardiac disease, pulmonary disease, malignant tumor	21	Cured
PA17	Urine	68/M	–	8	Cured
PA18	Urine	83/M	Cardiac disease, pulmonary disease, hepatobiliary disease, malignant tumor	4	Cured
PA19	Urine	34/F	Hepatobiliary disease, kidney disease	18	Cured
PA20	Urine	77/M	Cardiac disease	8	Cured
PA21	Urine	34/F	Pulmonary disease, hepatobiliary disease, kidney disease	27	Cured
PA22	Sputum	33/M	Diabetes, pulmonary disease, hepatobiliary disease	14	Cured
PA23	Sputum	73/F	Hypertension, pulmonary disease	8	Cured
PA24	Urine	57/M	Kidney disease	7	Cured
PA25	Urine	52/M	Hypertension, kidney disease	14	Cured
PA26	Urine	35/F	Hepatobiliary disease	15	Cured
PA27	Urine	28/F	Kidney disease	6	Cured
PA28	Urine	80/M	Diabetes, hypertension, malignant tumor	10	Cured
PA29	Urine	64/F	Kidney disease	14	Cured
PA30	Urine	64/F	Kidney disease	14	Cured
PA31	Urine	57/F	Hypertension, cardiac disease, malignant tumor	28	Cured
PA32	Urine	69/M	Malignant tumor	6	Cured

Automatic discharge, the patient gave up treatment due to serious illness conditions. LOS, length of hospital stay. -, negative.

**Table 2:** Strain characteristics and combined antimicrobial susceptibility tests results of XDR-PA strains.

ID	ST	Carbapenemase expression	Efflux pumps (*mexA*, *mexC*) overexpression	Virulence genes	ATM (MIC)	CZA (MIC)	ATM+CZA (MIC)	FIC
PA1	244	–	+	*exoY+/exoT+/exoU-/exoS+*	2	1/4	1/(0.25/4)	0.75
PA2	1203	–	+	*exoY-/exoT+/exoU+/exoS-*	8	2/4	4/(1/4)	1.00
PA3	810	–	+	*exoY+/exoT+/exoU-/exoS+*	≥ 64	≥ 128/4	16/(0.125/4)	0.25
PA4	1971	NDM-1	–	*exoY+/exoT+/exoU+/exoS-*	≥ 64	≥ 128/4	16/(0.125/4)	0.25
PA5	1971	NDM-1	+	*exoY+/exoT+/exoU+/exoS-*	≥ 64	≥ 128/4	16/(0.125/4)	0.25
PA6	1971	NDM-1	+	*exoY+/exoT+/exoU+/exoS-*	≥ 64	≥ 128/4	16/(0.125/4)	0.25
PA7	2326	–	+	*exoY+/exoT+/exoU+/exoS-*	8	2/4	0.25/(2/4)	0.56
PA8	308	NDM-1	–	*exoY+/exoT+/exoU+/exoS-*	≥ 64	≥ 128/4	16/(128/4)	1.25
PA9	1971	NDM-1	–	*exoY+/exoT+/exoU+/exoS-*	≥ 64	≥ 128/4	16/(0.125/4)	0.25
PA10	274	–	+	*exoY+/exoT+/exoU-/exoS+*	4	1/4	2/(0.125/4)	0.63
PA11	308	NDM-1	–	*exoY+/exoT+/exoU+/exoS-*	≥ 64	≥ 128/4	16/(128/4)	1.25
PA12	1971	NDM-1	–	*exoY+/exoT+/exoU+/exoS-*	≥ 64	≥ 128/4	16/(0.125/4)	0.25
PA13	1971	NDM-1	–	*exoY+/exoT+/exoU+/exoS-*	≥ 64	≥ 128/4	16/(0.125/4)	0.25
PA14	1971	NDM-1	–	*exoY+/exoT+/exoU+/exoS-*	≥ 64	≥ 128/4	16/(0.125/4)	0.25
PA15	1971	NDM-1	–	*exoY+/exoT+/exoU+/exoS-*	≥ 64	≥ 128/4	16/(0.125/4)	0.25
PA16	308	NDM-1	–	*exoY+/exoT+/exoU+/exoS-*	≥ 64	≥ 128/4	16/(0.125/4)	0.25
PA17	1971	NDM-1	–	*exoY+/exoT+/exoU+/exoS-*	≥ 64	≥ 128/4	16/(0.125/4)	0.25
PA18	1971	NDM-1	–	*exoY+/exoT+/exoU+/exoS-*	≥ 64	≥ 128/4	16/(0.125/4)	0.25
PA19	1971	NDM-1	–	*exoY+/exoT+/exoU+/exoS-*	≥ 64	≥ 128/4	16/(0.125/4)	0.25
PA20	1971	NDM-1	–	*exoY+/exoT+/exoU+/exoS-*	≥ 64	≥ 128/4	16/(0.125/4)	0.25
PA21	1971	NDM-1	–	*exoY+/exoT+/exoU+/exoS-*	≥ 64	≥ 128/4	16/(0.125/4)	0.25
PA22	1971	NDM-1	+	*exoY+/exoT+/exoU+/exoS-*	≥ 64	8/4	16/(0.125/4)	0.27
PA23	357	VIM-2	–	*exoY+/exoT+/exoU+/exoS-*	≥ 64	≥ 128/4	16/(0.125/4)	0.25
PA24	1971	NDM-1	+	*exoY+/exoT+/exoU+/exoS-*	≥ 64	≥ 128/4	16/(0.125/4)	0.25
PA25	1971	NDM-1	–	*exoY+/exoT+/exoU+/exoS-*	≥ 64	≥ 128/4	16/(128/4)	1.25
PA26	1971	NDM-1	–	*exoY+/exoT+/exoU+/exoS-*	≥ 64	≥ 128/4	16/(0.125/4)	0.25
PA27	1971	NDM-1	–	*exoY+/exoT+/exoU+/exoS-*	≥ 64	≥ 128/4	16/(0.125/4)	0.25
PA28	1971	NDM-1	–	*exoY+/exoT+/exoU+/exoS-*	≥ 64	≥ 128/4	16/(0.125/4)	0.25
PA29	1971	NDM-1	–	*exoY+/exoT+/exoU+/exoS-*	≥ 64	≥ 128/4	16/(128/4)	1.25
PA30	1971	NDM-1	–	*exoY+/exoT+/exoU+/exoS-*	≥ 64	≥ 128/4	16/(128/4)	1.25
PA31	1971	NDM-1	–	*exoY+/exoT+/exoU+/exoS-*	≥ 64	≥ 128/4	16/(0.125/4)	0.25
PA32	1971	NDM-1	+	*exoY+/exoT+/exoU+/exoS-*	≥ 64	≥ 128/4	16/(128/4)	1.25

FIC, fractional inhibitory concentration index; FIC ≤ 0.5, synergistic effect; 0.5 < FIC ≤ 1, additive effect; 1 < FIC ≤ 2, irrelevant effect; FIC > 2, antagonistic effect. *mexA* overexpression, *mexA* expression level in XDR-PA strains ≥ 3-fold of that in the PAO1 strain; *mexC* overexpression, *mexC* expression level in XDR-PA strains ≥ 10-fold of that in the PAO1 strain. ATM+CZA (MIC), the MIC value of ATM and CZA in combined antimicrobial susceptibility testing. -, negative; +, positive.

### Detection of carbapenemase resistance genes and virulence genes

Carbapenemase resistance genes detection results showed that 26 NDM-1 (81.25%, 26/32) and 1 VIM-2 (3.13%, 1/32) were identified. As shown in **Table 2**. The virulence genes carried by XDR-PA strains mainly included *exoY+/exoT+/exoU+/exoS-* (87.50%, 28/32), *exoY+/exoT+/exoU-/exoS+* (9.38%, 3/32) and *exoY-/exoT+/exoU+/exoS-* (3.13%, 1/32). As shown in **Table 2**.

### Efflux pump systems, *oprD2* and *ampC* detection

**Figure 1:**
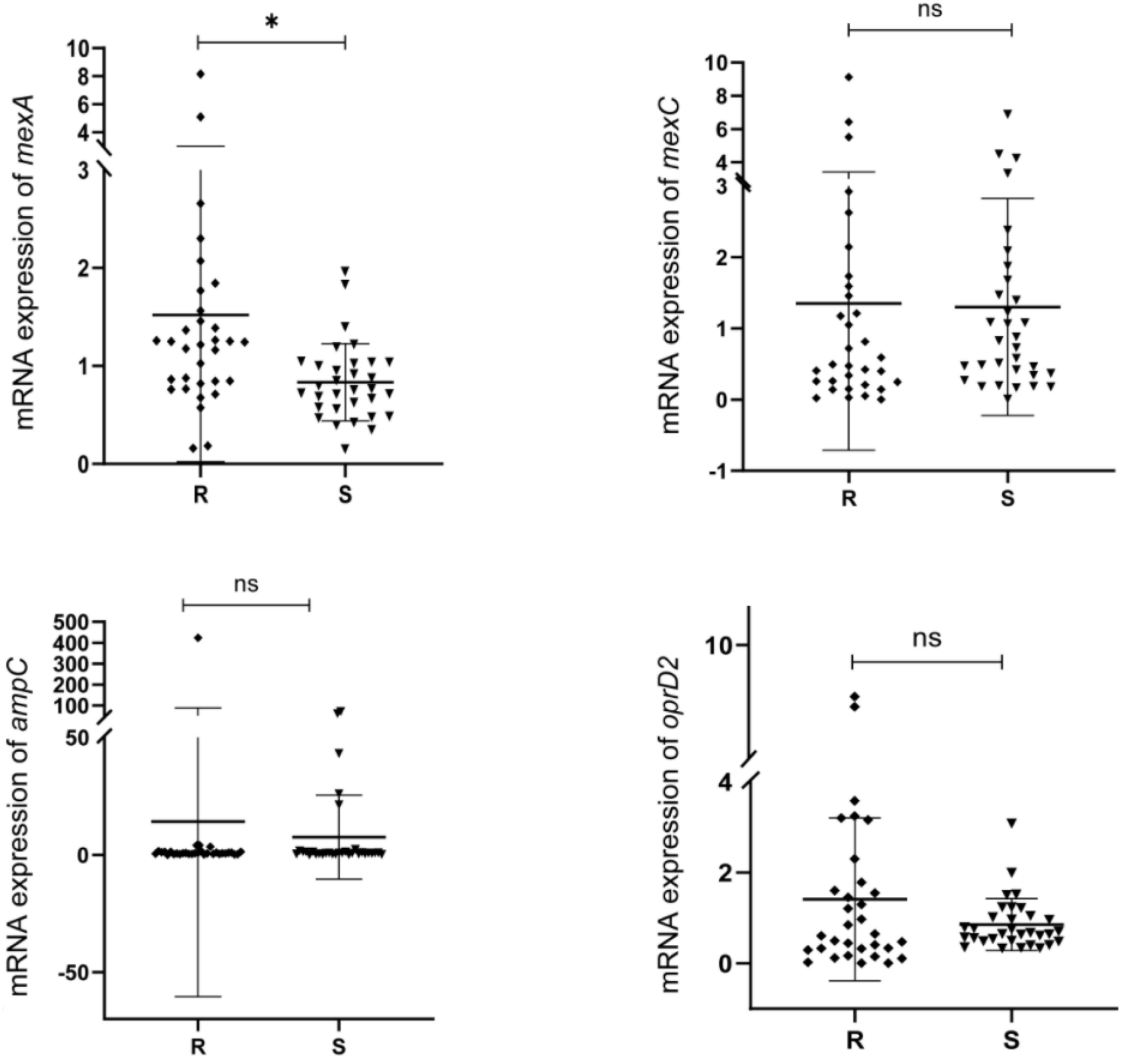
mRNA expression of *mexA, mexC, ampC* and *oprD2* in XDR-PA strains. R, XDR-PA group; S, S-PA group. ns, *P*>0.05; *P<0.05.

The qPCR test results revealed that the efflux pump *mexA* had a higher expression in the XDR-PA group than that in the S-PA group (*p* = 0.015), while no statistical difference was observed in the expression of *mexC*, *ampC* and *oprD2* between the two groups (*P* > 0.05). As shown in **Figure 1**.

### Multilocus sequence typing

A total of 8 different ST types were identified among the 32 XDR-PA strains, mainly including ST1971 and ST308, and the predominant ST type was ST1971 (71.88%, 23/32), with carrying both NDM-1 and *exoY+/exoT+/exoU+/exoS-*. As shown in **Table 2**.

### Combined antimicrobial susceptibility testing

Combined antimicrobial susceptibility testing showed that among the 27 CZA-resistant XDR-PA strains, CZA and ATM combination showed a synergistic effect on 21 CZA-resistant XDR-PA strains (77.78%, 21/27) (FIC ≤ 0.5), of which there were 20 strains (95.24%, 20/21) producing both MBL and *exoY+/exoT+/exoU+/exoS-*. As shown in **Table 2**.

## Discussion

Infections caused by XDR-PA have been increasingly reported worldwide, posing a significant threat to public health due to limited treatment options and high mortality rates (Montero et al., 2020). The resistance mechanisms of XDR-PA are multifactorial, involving a combination of intrinsic, acquired, and adaptive factors (Chevalier et al., 2017). In our study, the expressions of *mexA* were statistically different between the XDR-PA group and S-PA group, indicating that the overexpression of efflux pump may play an important role in XDR-PA phenotype. The efflux pump systems, which are widely present in microorganisms, can actively expel both metabolic wastes and antibiotics that inhibit bacterial growth (Lorusso et al., 2022). These systems exhibit a broad substrate spectrum and mediate resistance to most antibiotics, including aminoglycosides, fluoroquinolones, β-lactamase inhibitors, macrolides, and sulfonamides, thus their overexpression may contribute to extensive-drug resistance (Castanheira et al., 2019; Helmann et al., 2019). In this study, carbapenemase resistance genes were detected, and the results showed that the main resistance genes carried by XDR-PA strains were NDM-1 and VIM-2, indicating that MBL may play an important role in XDR phenotype. Previous studies have shown that NDM-1 was carried by various plasmids that co-contain multiple resistance genes, including carbapenemase, β-lactamase and fluoroquinolone resistance genes (Poirel et al., 2011; Jaidane et al., 2018; Politi et al., 2019; Shahin and Ahmadi, 2021), making NDM-1-carrying PA show resistance to most antibiotics, while remaining susceptible to only a few antibiotics such as polymyxin. Even though polymyxin demonstrates susceptibility *in vitro* and is regarded as an alternative for XDR-PA infections, its clinical use is restricted due to its narrow therapeutic window and toxicity (Motsch et al., 2020; Satlin, 2021). Thus, the optimal treatment for MBL-producing XDR-PA remains unclear. Novel antibiotic CZA has been approved for hospital-acquired pneumonia/ventilator-associated pneumonia, complex urinary tract infections and intra-abdominal infections (Carmeli et al., 2016). Avibactam (AVI) is a novel β-lactamase inhibitor, which can inhibit class A, class C and some class D (i.e., OXA-10 and OXA-48) β-lactamase, while it is not active against MBL-producing XDR-PA (Zhen et al., 2022). Therefore, it is urgent to find an effective therapy strategy for these XDR-PA strains. Although ATM remains stable against MBL-producing strains, it is usually inhibited by the frequent co-production of class A β-lactamases or *ampC*-type enzyme (Tan et al., 2021). Thus, the combination of ATM and CZA is considered as a potential treatment option for MBL-producing pathogens, as the combination will restore the activity of ATM against these strains attribute to the inhibition of the co-producing non-MBL β-lactamases by AVI (Mikhail et al., 2019; Cornely et al., 2020; Lee et al., 2021). However, clinical data on this combination is still scarce. Therefore, we performed the combined antimicrobial susceptibility tests of ATM and CZA on XDR-PA strains to provide some clues. The results revealed that the combination of ATM and CZA showed a synergistic effect on most MBL-producing XDR-PA strains, suggesting that the combination of ATM and CZA may be a potential treatment option for MBL-producing XDR-PA infections, which was consistent with some previous reports (Emeraud et al., 2019; Lee et al., 2021). Meanwhile, it is necessary to be vigilant against extensively drug-resistant mediated by KPC enzyme mutants or some D-class enzymes (i.e., OXA-14 and OXA-23), in which case the combination of ATM and CZA is usually ineffective.

T3SS, mainly includes *exoY*, *exoT*, *exoU* and *exoS*, play an important role in PA infection. PA transports a series of self-secretion proteins to host cells via the T3SS and destroys the actin skeleton and cause host cells death (Horna and Ruiz, 2021b; Moir et al., 2021). In our study, the T3SS virulence genes carried by XDR-PA strains mainly were *exoY+/exoT+/exoU+/exoS-*. Previous studies have reported that PA with *exoU+/exoS-* had a higher antibiotic resistance rate than that with *exoU-/exoS+*, may be because that PA with *exoU+/exoS-* frequently exhibiting overexpression of efflux pump (Takata et al., 2018; Ali, 2022). In addition, the strains carrying *exoU+/exoS-* exhibit high virulence, making their infection involve a wide range of lesions and increases their exposure to antibiotics, thus resulting in higher resistance rate (Takata et al., 2018; Ali, 2022). However, the relationship between antibiotic resistance and virulence remains controversial. Some researchers believe that the acquisition of resistance mechanisms may have a negative impact on adaptability (namely adaptability cost), resulting in bacteria physiological damage and loss of virulence (Gómez-Zorrilla et al., 2016). On the contrary, it has been reported that some resistance mutations are not associated with adaptive costs (Subedi et al., 2018). XDR-PA strains may develop other compensatory or inhibitory mutations that allow them to regain virulence without affecting resistance (Subedi et al., 2018).

Global clones associated with MDR/XDR-PA phenotypes are known as high-risk clones and pose great threat in hospitals around the world (Del Barrio-Tofiño et al., 2020). In our study, the 32 XDR-PA strains belong to 8 different ST types, and the predominant ST type was ST1971, with carrying both NDM-1 and *exoY+/exoT+/exoU+/exoS-*, suggesting that NDM-1-producing ST1971 XDR-PA with *exoY+/exoT+/exoU+/exoS-* was the predominant strain in our study. ST1971, a high-risk clone predominantly reported in China, is commonly associated with high virulence, it was first isolated in Beijing in 2015 and later in Guangdong in 2023 (Zhao et al., 2023a; Zhao et al., 2023b), which alarmed the further surveillance of this highly virulence and resistant clone. In addition, we also identified other high-risk global clones, including ST308, ST244 and ST357, which were recognized as the top 10 high-risk global clones (Del Barrio-Tofiño et al., 2020). Therefore, it is essential to implement effective infection control measures to prevent the spread of these high-risk clones.

Our study revealed the resistance mechanisms, molecular epidemiology and virulence genes of XDR-PA strains, as well as the synergistic activity of ATM and CZA combination against XDR-PA. The main limitation is that no *in vivo* validation was included in our study. In addition, the genes detected are limited compared with whole-genome sequencing. More studies on the mechanisms of synergistic activity between ATM and CZA combination as well as *in vivo* validation will be performed in subsequent research.

## Conclusions

In summary, the underlying resistance mechanisms of XDR-PA involved the overexpression of efflux pump *mexA* and the existence of MBL. In addition, ST1971 was the predominant ST type in our study, with carrying both NDM-1 and *exoY+/exoT+/exoU+/exoS-*. Furthermore, combined antimicrobial susceptibility testing suggested that CZA and ATM combination has potential against MBL-producing *exoY+/exoT+/exoU+/exoS-* XDR-PA. These findings may provide clues for the prevention, control and treatment strategies of XDR-PA infections.

## Data Availability

The raw data supporting the conclusions of this article will be made available by the authors, without undue reservation.

## Supplementary material

Supplementary Table 1 Antimicrobial susceptibility tests results of XDR-PA strains (MIC, μg/mL).
