# Supplementary material for: Synergistic activity of ceftazidime/avibactam combined with aztreonam against MBL-producing exoY+/exoT+/exoU+/exoS- extensively drug-resistant Pseudomonas aeruginosa
Source: Front Cell Infect Microbiol. 2026 Feb 27;16:1737414. doi: 10.3389/fcimb.2026.1737414 (PMC12982442; doi:10.3389/fcimb.2026.1737414)
Supplement: Supplementary Table 1 — Antimicrobial susceptibility tests results of XDR-PA strains (MIC, μg/mL). [file Table1.pdf]

Supplementary Table S1 Antimicrobial susceptibility tests results of XDR-PA strains (MIC, μg/mL)

ID	Ceftazidime	Cefepime	Ticarcillin/ clavulanic acid	Piperacillin/ Tazobactam	Cefoperazone/ Sulbactam	Levofloxacin	Ciprofloxacin	Imipenem	Meropenem	Aztreonam	Amikacin	Tobramycin	Polymyxin
P1	≥64 (R)	≥32 (R)	≥128 (R)	≥128 (R)	≥64 (R)	≥8 (R)	≥4 (R)	≥16 (R)	≥16 (R)	2 (S)	≥64 (R)	≥16 (R)	2 (S)
P2	≥64 (R)	≥32 (R)	≥128 (R)	6 (mm) (R)	≥64 (R)	≥8 (R)	≥4 (R)	6 (mm) (R)	≥16 (R)	8 (S)	≥64 (R)	≥16 (R)	≤0.5 (S)
P3	≥64 (R)	≥32 (R)	≥128 (R)	≥128 (R)	≥64 (R)	≥8 (R)	≥4 (R)	≥16 (R)	≥16 (R)	≥64 (R)	≥64 (R)	≥16 (R)	≤0.5 (S)
P4	≥64 (R)	≥32 (R)	≥128 (R)	≥128 (R)	≥64 (R)	≥8 (R)	≥4 (R)	≥16 (R)	≥16 (R)	≥64 (R)	≥64 (R)	≥16 (R)	≤0.5 (S)
P5	≥64 (R)	≥32 (R)	≥128 (R)	≥128 (R)	32 (I)	≥8 (R)	≥4 (R)	≥16 (R)	≥16 (R)	≥64 (R)	≥64 (R)	≥16 (R)	≤0.5 (S)
P6	≥64 (R)	≥32 (R)	≥128 (R)	≥128 (R)	≥64 (R)	≥8 (R)	≥4 (R)	≥16 (R)	≥16 (R)	≥64 (R)	≥64 (R)	≥16 (R)	2 (S)
P7	≥64 (R)	≥32 (R)	≥128 (R)	≥128 (R)	≥64 (R)	≥8 (R)	≥4 (R)	≥16 (R)	≥16 (R)	8 (S)	≥64 (R)	≥16 (R)	1 (S)
P8	≥64 (R)	≥32 (R)	≥128 (R)	≥128 (R)	≥64 (R)	≥8 (R)	≥4 (R)	≥16 (R)	≥16 (R)	≥64 (R)	≥64 (R)	≥16 (R)	≤0.5 (S)
P9	≥64 (R)	≥32 (R)	≥128 (R)	≥128 (R)	≥64 (R)	≥8 (R)	≥4 (R)	≥16 (R)	≥16 (R)	≥64 (R)	≥64 (R)	≥16 (R)	2 (S)
P10	≥64 (R)	≥32 (R)	≥128 (R)	≥128 (R)	≥64 (R)	≥8 (R)	6 (mm) (R)	≥16 (R)	6 (mm) (R)	4 (S)	≥64 (R)	≥16 (R)	4 (R)
P11	≥64 (R)	≥32 (R)	≥128 (R)	≥128 (R)	≥64 (R)	≥8 (R)	≥4 (R)	≥16 (R)	≥16 (R)	≥64 (R)	≥64 (R)	≥16 (R)	≤0.5 (S)
P12	≥64 (R)	≥32 (R)	≥128 (R)	9 (mm) (R)	≥64 (R)	≥8 (R)	≥4 (R)	6 (mm) (R)	≥16 (R)	≥64 (R)	≥64 (R)	≥16 (R)	2 (S)
P13	≥64 (R)	≥32 (R)	≥128 (R)	≥128 (R)	≥64 (R)	≥8 (R)	≥4 (R)	≥16 (R)	≥16 (R)	≥64 (R)	≥64 (R)	≥16 (R)	4 (R)
P14	≥64 (R)	≥32 (R)	≥128 (R)	≥128 (R)	≥64 (R)	≥8 (R)	≥4 (R)	≥16 (R)	≥16 (R)	≥64 (R)	≥64 (R)	≥16 (R)	2 (S)
P15	≥64 (R)	≥32 (R)	≥128 (R)	≥128 (R)	≥64 (R)	≥8 (R)	≥4 (R)	≥16 (R)	≥16 (R)	≥64 (R)	≥64 (R)	≥16 (R)	2 (S)
P16	≥64 (R)	≥32 (R)	≥128 (R)	≥128 (R)	≥64 (R)	≥8 (R)	≥4 (R)	≥16 (R)	≥16 (R)	≥64 (R)	≥64 (R)	≥16 (R)	≤0.5 (S)
P17	≥64 (R)	≥32 (R)	≥128 (R)	≥128 (R)	≥64 (R)	≥8 (R)	≥4 (R)	≥16 (R)	≥16 (R)	≥64 (R)	≥64 (R)	≥16 (R)	2 (S)
P18	≥64 (R)	≥32 (R)	≥128 (R)	9 (mm) (R)	≥64 (R)	≥8 (R)	≥4 (R)	6 (mm) (R)	≥16 (R)	≥64 (R)	≥64 (R)	≥16 (R)	2 (S)
P19	≥64 (R)	≥32 (R)	≥128 (R)	≥128 (R)	≥64 (R)	≥8 (R)	≥4 (R)	≥16 (R)	≥16 (R)	≥64 (R)	≥64 (R)	≥16 (R)	≤0.5 (S)
P20	≥64 (R)	≥32 (R)	≥128 (R)	≥128 (R)	≥64 (R)	≥8 (R)	≥4 (R)	≥16 (R)	≥16 (R)	≥64 (R)	≥64 (R)	≥16 (R)	≤0.5 (S)
P21	≥64 (R)	≥32 (R)	≥128 (R)	≥128 (R)	≥64 (R)	≥8 (R)	≥4 (R)	≥16 (R)	≥16 (R)	≥64 (R)	≥64 (R)	≥16 (R)	≤0.5 (S)
P22	≥64 (R)	≥32 (R)	≥128 (R)	7 (mm) (R)	≥64 (R)	≥8 (R)	≥4 (R)	9 (mm) (R)	≥16 (R)	≥64 (R)	32 (I)	≥16 (R)	≤0.5 (S)
P23	6 (mm) (R)	6 (mm) (R)	≥128 (R)	6 (mm) (R)	6 (mm) (R)	6 (mm) (R)	6 (mm) (R)	6 (mm) (R)	6 (mm) (R)	≥64 (R)	7 (mm)(R)	≥16 (R)	≤0.5 (S)
P24	≥64 (R)	≥32 (R)	≥128 (R)	6 (mm) (R)	≥64 (R)	≥8 (R)	≥4 (R)	6 (mm) (R)	≥16 (R)	≥64 (R)	≥64 (R)	≥16 (R)	≤0.5 (S)
P25	≥64 (R)	≥32 (R)	≥128 (R)	6 (mm) (R)	≥64 (R)	≥8 (R)	≥4 (R)	6 (mm) (R)	≥16 (R)	≥64 (R)	≥64 (R)	≥16 (R)	≤0.5 (S)
P26	≥64 (R)	≥32 (R)	≥128 (R)	6 (mm) (R)	≥64 (R)	≥8 (R)	≥4 (R)	≥16 (R)	≥16 (R)	≥64 (R)	≥64 (R)	≥16 (R)	≤0.5 (S)
P27	≥64 (R)	≥32 (R)	≥128 (R)	≥128 (R)	≥64 (R)	≥8 (R)	≥4 (R)	≥16 (R)	≥16 (R)	≥64 (R)	≥64 (R)	≥16 (R)	≤0.5 (S)
P28	≥64 (R)	≥32 (R)	≥128 (R)	≥128 (R)	≥64 (R)	≥8 (R)	≥4 (R)	≥16 (R)	≥16 (R)	≥64 (R)	≥64 (R)	≥16 (R)	≤0.5 (S)
P29	≥64 (R)	≥32 (R)	≥128 (R)	≥128 (R)	≥64 (R)	≥8 (R)	≥4 (R)	≥16 (R)	≥16 (R)	≥64 (R)	≥64 (R)	≥16 (R)	≤0.5 (S)
P30	≥64 (R)	≥32 (R)	≥128 (R)	≥128 (R)	≥64 (R)	≥8 (R)	≥4 (R)	≥16 (R)	≥16 (R)	≥64 (R)	≥64 (R)	≥16 (R)	≤0.5 (S)
P31	≥64 (R)	≥32 (R)	≥128 (R)	≥128 (R)	≥64 (R)	≥8 (R)	≥4 (R)	≥16 (R)	≥16 (R)	≥64 (R)	≥64 (R)	≥16 (R)	≤0.5 (S)
P32	≥64 (R)	≥32 (R)	≥128 (R)	6 (mm) (R)	≥64 (R)	≥8 (R)	≥4 (R)	6 (mm) (R)	≥16 (R)	≥64 (R)	≥64 (R)	≥16 (R)	≤0.5 (S)

R, resistant; S, susceptible; I, intermediate. mm, the diameter of inhibition zone by Kirby–Bauer disk diffusion method.
